# *rac*-2,2′-(Thiane-2,6-di­yl)bis­[1-(4-bromo­phen­yl)ethanone]

**DOI:** 10.1107/S1600536811011792

**Published:** 2011-04-07

**Authors:** Li-Qiang Liu, Jing-Kui Yang

**Affiliations:** a College of Chemistry and Chemical Engineering, Graduate University of Chinese, Academy of Sciences, Beijing, 100049, People’s Republic of China

## Abstract

In the title compound, C_21_H_20_Br_2_O_2_S, prepared by the reaction of 1,9-bis­(4-bromo­phen­yl)nona-2,7-diene-1,9-dione with sodium sulfide nona­hydrate in acetonitrile, the six-membered thio­pyran ring has a chair conformation while the H atoms *ortho* to the S atom adopt a *cis* configuration. The dihedral angle between the two benzene rings is 2.59 (8)°.

## Related literature

For the synthesis of 1,9-bis­(4-bromo­phen­yl)nona-2,7-diene-1,9-dione, see: Yang, Cauble *et al.* (2004 ▶); Yang, Felton *et al.* (2004 ▶). For the synthesis of compounds containing sulfur, see: Knapp *et al.* (2002 ▶); Yao *et al.* (2003 ▶); Oliveira *et al.* (1999 ▶). For applications of natural products containing sulfur, see: Qi *et al.* (2004 ▶); Zhang & Zhang (2006 ▶); Barco *et al.* (2006 ▶).
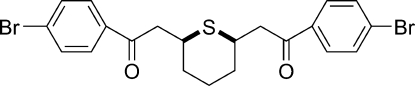


## Experimental

### 

#### Crystal data

C_21_H_20_Br_2_O_2_S
*M* *_r_* = 496.25
Triclinic, 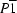

*a* = 6.484 (4) Å
*b* = 12.970 (5) Å
*c* = 13.076 (4) Å
α = 71.14 (3)°
β = 79.45 (4)°
γ = 79.52 (4)°
*V* = 1014.1 (7) Å^3^
*Z* = 2
Mo *K*α radiation
μ = 4.11 mm^−1^
*T* = 295 K
0.40 × 0.30 × 0.20 mm

#### Data collection

Siemens P4 four-circle diffractometer
Absorption correction: ψ scan (North *et al.*, 1968 ▶) *T*_min_ = 0.381, *T*_max_ = 0.459
4549 measured reflections
3548 independent reflections
2220 reflections with *I* > 2σ(*I*)
*R*_int_ = 0.032
3 standard reflections every 97 reflections  intensity decay: none

#### Refinement

*R*[*F* ^2^ > 2σ(*F* ^2^)] = 0.055
*wR*(*F* ^2^) = 0.112
*S* = 1.07
3548 reflections
235 parameters
H-atom parameters constrained
Δρ_max_ = 0.51 e Å^−3^
Δρ_min_ = −0.41 e Å^−3^

### 

Data collection: *XSCANS* (Bruker, 1997 ▶); cell refinement: *XSCANS*; data reduction: *XSCANS*; program(s) used to solve structure: *SHELXS97* (Sheldrick, 2008 ▶); program(s) used to refine structure: *SHELXL97* (Sheldrick, 2008 ▶); molecular graphics: *SHELXTL* (Sheldrick, 2008 ▶); software used to prepare material for publication: *SHELXTL*.

## Supplementary Material

Crystal structure: contains datablocks I, global. DOI: 10.1107/S1600536811011792/zs2103sup1.cif

Structure factors: contains datablocks I. DOI: 10.1107/S1600536811011792/zs2103Isup2.hkl

Additional supplementary materials:  crystallographic information; 3D view; checkCIF report

**Fig. 1.**
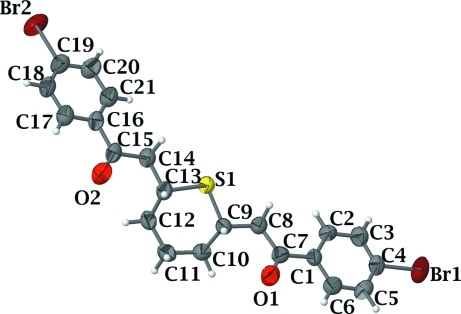
The molecular conformation of the title compound showing 35% probability displacement ellipsoids and the atom numbering scheme

Enhanced figure: interactive version of Fig. 1

## supplementary crystallographic information

### Comment

The mothods for the synthesis of compounds containing sulfur have been described (Knapp *et al.*, 2002; Yao *et al.*, 2003; Oliveira *et al.* 1999). Natural products containing sulfur often have biological activity, so that the methods for their synthesis have received considerable attention among researchers. In this paper, we report the structure of the title compound C_21_H_20_Br_2_O_2_S (I), prepared by the reaction of 1,9-bis(4-bromophenyl)nona-2,7-diene-1,9-dione with sodium sulfide nonahydrate in acetonitrile. In the crystal, the six-membered thiopyran ring has a chair conformation with the H atoms *ortho* to the S (H9 and H13) adopting a *cis* configuration (Fig. 1). The dihedral angle between the benzene rings on the substituent chains is 2.59 (8)°.

### Experimental

The reaction mixture of 1,9-bis(4-bromophenyl)nona-2,7-diene-1,9-dione (100 mg, 0.20 mmol) with sodium sulfide nonahydrate (67 mg, 0.28 mmol) in 50 ml of acetonitrile was stirred for 11 days at room temperature, affording the title compound (20 mg; yield 32%). Colorless single crystals were obtained by slow evaporation of an ethyl acetate solution in air.

### Refinement

All hydrogen atoms were generated geometically with C—H bond distances of 0.93–0.98 Å according to criteria described in the *SHELXTL* manual (Bruker, 1997). These were included in the refinement with *U*_iso_(H) = 1.2*U*_eq_(C).

### Crystal data

**Table:**

C_21_H_20_Br_2_O_2_S	*Z* = 2
*M**_r_* = 496.25	*F*(000) = 496
Triclinic, *P*1	*D*_x_ = 1.625 Mg m^−^^3^
Hall symbol: -P 1	Mo *K*α radiation, λ = 0.71073 Å
*a* = 6.484 (4) Å	Cell parameters from 45 reflections
*b* = 12.970 (5) Å	θ = 5.6–12.4°
*c* = 13.076 (4) Å	µ = 4.11 mm^−^^1^
α = 71.14 (3)°	*T* = 295 K
β = 79.45 (4)°	Prism, colorless
γ = 79.52 (4)°	0.40 × 0.30 × 0.20 mm
*V* = 1014.1 (7) Å^3^	

### Data collection

**Table:**

Siemens P4 four-circle diffractometer	2220 reflections with *I* > 2σ(*I*)
Radiation source: fine-focus sealed tube	*R*_int_ = 0.032
graphite	θ_max_ = 25.1°, θ_min_ = 2.0°
ω scans	*h* = −1→7
Absorption correction: ψ scan (North *et al.*, 1968)	*k* = −14→14
*T*_min_ = 0.381, *T*_max_ = 0.459	*l* = −15→15
4549 measured reflections	3 standard reflections every 97 reflections
3548 independent reflections	intensity decay: none

### Refinement

**Table:**

Refinement on *F*^2^	Primary atom site location: structure-invariant direct methods
Least-squares matrix: full	Secondary atom site location: difference Fourier map
*R*[*F*^2^ > 2σ(*F*^2^)] = 0.055	Hydrogen site location: inferred from neighbouring sites
*wR*(*F*^2^) = 0.112	H-atom parameters constrained
*S* = 1.07	*w* = 1/[σ^2^(*F*_o_^2^) + (0.001*P*)^2^ + 2.0*P*] where *P* = (*F*_o_^2^ + 2*F*_c_^2^)/3
3548 reflections	(Δ/σ)_max_ = 0.001
235 parameters	Δρ_max_ = 0.51 e Å^−^^3^
0 restraints	Δρ_min_ = −0.41 e Å^−^^3^

### Special details

**Table:**

Geometry. All e.s.d.'s (except the e.s.d. in the dihedral angle between two l.s. planes) are estimated using the full covariance matrix. The cell e.s.d.'s are taken into account individually in the estimation of e.s.d.'s in distances, angles and torsion angles; correlations between e.s.d.'s in cell parameters are only used when they are defined by crystal symmetry. An approximate (isotropic) treatment of cell e.s.d.'s is used for estimating e.s.d.'s involving l.s. planes.
Refinement. Refinement of *F*^2^ against ALL reflections. The weighted *R*-factor *wR* and goodness of fit *S* are based on *F*^2^, conventional *R*-factors *R* are based on *F*, with *F* set to zero for negative *F*^2^. The threshold expression of *F*^2^ > σ(*F*^2^) is used only for calculating *R*-factors(gt) *etc*. and is not relevant to the choice of reflections for refinement. *R*-factors based on *F*^2^ are statistically about twice as large as those based on *F*, and *R*- factors based on ALL data will be even larger.

### Fractional atomic coordinates and isotropic or equivalent isotropic displacement parameters (Å^2^)

**Table:**

	*x*	*y*	*z*	*U*_iso_*/*U*_eq_	
Br1	0.47955 (9)	1.39832 (4)	0.65329 (4)	0.08336 (16)	
Br2	0.87756 (10)	0.46636 (4)	−0.19734 (4)	0.09195 (19)	
S1	0.35503 (17)	0.97967 (9)	0.19248 (8)	0.0594 (3)	
O1	−0.0519 (5)	1.2370 (2)	0.3377 (2)	0.0755 (9)	
O2	0.0963 (5)	0.8256 (2)	−0.0195 (2)	0.0740 (9)	
C1	0.2190 (6)	1.2490 (3)	0.4300 (3)	0.0477 (10)	
C2	0.4267 (6)	1.2193 (3)	0.4523 (3)	0.0596 (12)	
H2A	0.5153	1.1687	0.4220	0.071*	
C3	0.5051 (7)	1.2633 (3)	0.5185 (3)	0.0673 (13)	
H3A	0.6453	1.2438	0.5316	0.081*	
C4	0.3711 (6)	1.3365 (3)	0.5645 (3)	0.0559 (11)	
C5	0.1643 (7)	1.3678 (3)	0.5447 (3)	0.0597 (12)	
H5A	0.0760	1.4175	0.5764	0.072*	
C6	0.0894 (7)	1.3245 (3)	0.4772 (3)	0.0568 (12)	
H6A	−0.0499	1.3460	0.4630	0.068*	
C7	0.1259 (7)	1.2030 (3)	0.3601 (3)	0.0543 (11)	
C8	0.2614 (6)	1.1124 (3)	0.3196 (3)	0.0557 (11)	
H8A	0.3138	1.0552	0.3815	0.067*	
H8B	0.3829	1.1420	0.2711	0.067*	
C9	0.1493 (6)	1.0605 (3)	0.2598 (3)	0.0494 (10)	
H9A	0.0776	1.1190	0.2042	0.059*	
C10	−0.0111 (6)	0.9885 (3)	0.3336 (3)	0.0601 (12)	
H10A	−0.1162	1.0322	0.3709	0.072*	
H10B	0.0599	0.9303	0.3884	0.072*	
C11	−0.1229 (6)	0.9372 (3)	0.2727 (3)	0.0650 (13)	
H11A	−0.2286	0.8956	0.3238	0.078*	
H11B	−0.1954	0.9953	0.2184	0.078*	
C12	0.0287 (6)	0.8618 (3)	0.2168 (3)	0.0588 (12)	
H12A	−0.0519	0.8293	0.1826	0.071*	
H12B	0.0979	0.8027	0.2717	0.071*	
C13	0.1970 (6)	0.9190 (3)	0.1311 (3)	0.0517 (11)	
H13A	0.1284	0.9771	0.0739	0.062*	
C14	0.3569 (6)	0.8426 (3)	0.0794 (3)	0.0561 (12)	
H14A	0.4755	0.8815	0.0400	0.067*	
H14B	0.4100	0.7805	0.1374	0.067*	
C15	0.2757 (7)	0.7987 (3)	0.0020 (3)	0.0543 (12)	
C16	0.4289 (6)	0.7209 (3)	−0.0478 (3)	0.0530 (11)	
C17	0.3596 (7)	0.6792 (3)	−0.1196 (3)	0.0645 (13)	
H17A	0.2225	0.7017	−0.1372	0.077*	
C18	0.4938 (7)	0.6047 (3)	−0.1650 (3)	0.0667 (13)	
H18A	0.4479	0.5780	−0.2137	0.080*	
C19	0.6922 (7)	0.5709 (3)	−0.1379 (3)	0.0646 (13)	
C20	0.7680 (8)	0.6127 (3)	−0.0700 (3)	0.0719 (14)	
H20A	0.9069	0.5912	−0.0550	0.086*	
C21	0.6352 (7)	0.6872 (3)	−0.0241 (3)	0.0657 (13)	
H21A	0.6846	0.7148	0.0229	0.079*	

### Atomic displacement parameters (Å^2^)

**Table:**

	*U*^11^	*U*^22^	*U*^33^	*U*^12^	*U*^13^	*U*^23^
Br1	0.1169 (4)	0.0718 (3)	0.0768 (3)	−0.0107 (3)	−0.0346 (3)	−0.0323 (2)
Br2	0.1217 (5)	0.0730 (3)	0.0745 (3)	0.0071 (3)	0.0042 (3)	−0.0334 (2)
S1	0.0517 (6)	0.0715 (6)	0.0667 (6)	−0.0140 (5)	−0.0006 (5)	−0.0374 (5)
O1	0.0630 (18)	0.0824 (17)	0.0972 (18)	0.0060 (16)	−0.0260 (15)	−0.0490 (15)
O2	0.0769 (19)	0.0751 (17)	0.0793 (17)	0.0154 (16)	−0.0348 (15)	−0.0364 (14)
C1	0.051 (2)	0.0488 (19)	0.0465 (19)	−0.0129 (18)	−0.0055 (17)	−0.0160 (16)
C2	0.049 (2)	0.067 (2)	0.067 (2)	−0.004 (2)	0.000 (2)	−0.0335 (19)
C3	0.063 (3)	0.075 (3)	0.074 (3)	−0.010 (2)	−0.014 (2)	−0.033 (2)
C4	0.070 (3)	0.053 (2)	0.050 (2)	−0.016 (2)	−0.013 (2)	−0.0154 (17)
C5	0.076 (3)	0.050 (2)	0.052 (2)	0.003 (2)	−0.011 (2)	−0.0193 (17)
C6	0.058 (3)	0.055 (2)	0.056 (2)	−0.001 (2)	−0.012 (2)	−0.0159 (18)
C7	0.055 (2)	0.055 (2)	0.053 (2)	−0.007 (2)	−0.0016 (19)	−0.0191 (17)
C8	0.056 (2)	0.060 (2)	0.058 (2)	−0.013 (2)	−0.0022 (19)	−0.0272 (17)
C9	0.043 (2)	0.057 (2)	0.0519 (19)	−0.0110 (18)	−0.0026 (17)	−0.0212 (17)
C10	0.056 (2)	0.064 (2)	0.061 (2)	−0.012 (2)	0.004 (2)	−0.0246 (19)
C11	0.052 (2)	0.070 (3)	0.072 (3)	−0.017 (2)	0.000 (2)	−0.019 (2)
C12	0.063 (3)	0.058 (2)	0.059 (2)	−0.024 (2)	−0.005 (2)	−0.0159 (19)
C13	0.057 (2)	0.051 (2)	0.0507 (19)	−0.0033 (19)	−0.0162 (18)	−0.0179 (16)
C14	0.063 (3)	0.055 (2)	0.055 (2)	−0.010 (2)	−0.011 (2)	−0.0196 (18)
C15	0.072 (3)	0.0383 (19)	0.052 (2)	−0.006 (2)	−0.013 (2)	−0.0118 (16)
C16	0.066 (3)	0.0443 (19)	0.051 (2)	−0.0110 (19)	−0.0120 (19)	−0.0125 (17)
C17	0.074 (3)	0.065 (2)	0.060 (2)	−0.010 (2)	−0.016 (2)	−0.0232 (19)
C18	0.080 (3)	0.068 (2)	0.064 (2)	−0.016 (2)	−0.011 (2)	−0.0317 (19)
C19	0.083 (3)	0.054 (2)	0.053 (2)	−0.007 (2)	0.001 (2)	−0.0183 (19)
C20	0.078 (3)	0.063 (2)	0.074 (3)	0.004 (2)	−0.013 (2)	−0.026 (2)
C21	0.075 (3)	0.068 (2)	0.061 (2)	−0.007 (2)	−0.015 (2)	−0.0262 (19)

### Geometric parameters (Å, °)

**Table:**

Br1—C4	1.895 (4)	C10—H10A	0.9700
Br2—C19	1.906 (4)	C10—H10B	0.9700
S1—C9	1.819 (4)	C11—C12	1.518 (6)
S1—C13	1.820 (4)	C11—H11A	0.9700
O1—C7	1.210 (5)	C11—H11B	0.9700
O2—C15	1.209 (5)	C12—C13	1.518 (5)
C1—C2	1.389 (5)	C12—H12A	0.9700
C1—C6	1.395 (5)	C12—H12B	0.9700
C1—C7	1.497 (6)	C13—C14	1.523 (5)
C2—C3	1.386 (6)	C13—H13A	0.9800
C2—H2A	0.9300	C14—C15	1.517 (6)
C3—C4	1.377 (6)	C14—H14A	0.9700
C3—H3A	0.9300	C14—H14B	0.9700
C4—C5	1.375 (6)	C15—C16	1.500 (5)
C5—C6	1.381 (6)	C16—C21	1.389 (6)
C5—H5A	0.9300	C16—C17	1.396 (6)
C6—H6A	0.9300	C17—C18	1.385 (6)
C7—C8	1.513 (5)	C17—H17A	0.9300
C8—C9	1.523 (6)	C18—C19	1.354 (6)
C8—H8A	0.9700	C18—H18A	0.9300
C8—H8B	0.9700	C19—C20	1.376 (7)
C9—C10	1.512 (5)	C20—C21	1.385 (6)
C9—H9A	0.9800	C20—H20A	0.9300
C10—C11	1.528 (6)	C21—H21A	0.9300
			
C9—S1—C13	101.01 (18)	C12—C11—H11B	109.1
C2—C1—C6	117.9 (4)	C10—C11—H11B	109.1
C2—C1—C7	123.7 (4)	H11A—C11—H11B	107.8
C6—C1—C7	118.4 (4)	C11—C12—C13	114.0 (3)
C3—C2—C1	121.6 (4)	C11—C12—H12A	108.8
C3—C2—H2A	119.2	C13—C12—H12A	108.8
C1—C2—H2A	119.2	C11—C12—H12B	108.8
C4—C3—C2	118.6 (4)	C13—C12—H12B	108.8
C4—C3—H3A	120.7	H12A—C12—H12B	107.7
C2—C3—H3A	120.7	C12—C13—C14	114.1 (3)
C5—C4—C3	121.6 (4)	C12—C13—S1	110.2 (3)
C5—C4—Br1	119.8 (3)	C14—C13—S1	104.6 (3)
C3—C4—Br1	118.6 (3)	C12—C13—H13A	109.2
C4—C5—C6	119.1 (4)	C14—C13—H13A	109.2
C4—C5—H5A	120.5	S1—C13—H13A	109.2
C6—C5—H5A	120.5	C15—C14—C13	116.0 (3)
C5—C6—C1	121.2 (4)	C15—C14—H14A	108.3
C5—C6—H6A	119.4	C13—C14—H14A	108.3
C1—C6—H6A	119.4	C15—C14—H14B	108.3
O1—C7—C1	120.8 (4)	C13—C14—H14B	108.3
O1—C7—C8	121.6 (4)	H14A—C14—H14B	107.4
C1—C7—C8	117.6 (3)	O2—C15—C16	121.3 (4)
C7—C8—C9	114.9 (3)	O2—C15—C14	121.8 (4)
C7—C8—H8A	108.5	C16—C15—C14	117.0 (4)
C9—C8—H8A	108.5	C21—C16—C17	118.6 (4)
C7—C8—H8B	108.5	C21—C16—C15	122.9 (4)
C9—C8—H8B	108.5	C17—C16—C15	118.5 (4)
H8A—C8—H8B	107.5	C18—C17—C16	120.4 (4)
C10—C9—C8	113.7 (3)	C18—C17—H17A	119.8
C10—C9—S1	110.1 (3)	C16—C17—H17A	119.8
C8—C9—S1	106.4 (3)	C19—C18—C17	119.6 (4)
C10—C9—H9A	108.8	C19—C18—H18A	120.2
C8—C9—H9A	108.8	C17—C18—H18A	120.2
S1—C9—H9A	108.8	C18—C19—C20	121.5 (4)
C9—C10—C11	113.2 (3)	C18—C19—Br2	120.3 (4)
C9—C10—H10A	108.9	C20—C19—Br2	118.1 (4)
C11—C10—H10A	108.9	C19—C20—C21	119.3 (5)
C9—C10—H10B	108.9	C19—C20—H20A	120.4
C11—C10—H10B	108.9	C21—C20—H20A	120.4
H10A—C10—H10B	107.7	C20—C21—C16	120.5 (4)
C12—C11—C10	112.6 (3)	C20—C21—H21A	119.8
C12—C11—H11A	109.1	C16—C21—H21A	119.8
C10—C11—H11A	109.1		
